# Rutoside decreases human macrophage-derived inflammatory mediators and improves clinical signs in adjuvant-induced arthritis

**DOI:** 10.1186/ar2372

**Published:** 2008-02-06

**Authors:** Tina Kauss, Daniel Moynet, Jérôme Rambert, Abir Al-Kharrat, Stephane Brajot, Denis Thiolat, Rachid Ennemany, Fawaz Fawaz, M Djavad Mossalayi

**Affiliations:** 1 Department of Immunology and Parasitology, EA3677, School of Pharmacy, Bordeaux 2 University, 146 rue Léo Saignat, 33076 Bordeaux, France; 2 Department of Galenic and Biopharmaceutics, EA3677, School of Pharmacy, Bordeaux 2 University, 146 rue Léo Saignat, 33076 Bordeaux, France; 3 Eurotest, 147 avenue de la Somme, 33700 Merignac, France

## Abstract

**Background:**

Dietary flavonols may play an important role in the adjunct therapy of chronic inflammation. The availability of therapeutic formulations of pentahydroxyflavone glycoside, rutoside (RU), led us to investigate the ability of this molecule to modulate the release of various proinflammatory mediators from human activated macrophages *in vitro *and to ameliorate arthritic markers in a rat model.

**Methods:**

RU was added simultaneously to human macrophages during their activation. Cells were then analyzed for inflammation-related gene expression using a specific array, and cell supernatants were collected to measure inflammatory mediators. RU was also injected into adjuvant-induced arthritic rats, and disease progression and body weight were evaluated until 50 days after injection. Sera and peritoneal macrophages were also collected to quantify the RU effect on various inflammatory markers.

**Results:**

RU inhibited inflammation-related gene expression in activated human macrophages and the release of nitric oxide, tumor necrosis factor-alpha, interleukin (IL)-1, and IL-6 from these cells. In a rat model, RU inhibited clinical signs of chronic arthritis, correlating with decreased levels of inflammatory cytokines detected in rat sera and macrophage supernatants.

**Conclusion:**

Thus, RU may have clinical value in reducing inflammatory manifestations in human arthritis and other inflammatory diseases.

## Introduction

The immune system has evolved to protect the host from microbial infection. Nevertheless, a breakdown in the immune system often results in infection, cancer, and autoimmune diseases. Multiple sclerosis, rheumatoid arthritis (RA), type 1 diabetes, inflammatory bowel disease, myocarditis, thyroiditis, uveitis, systemic lupus erythromatosis, and myasthenia gravis are organ-specific autoimmune diseases that afflict more than 5% of the population worldwide. Although their etiology is not known and a cure is still wanting, promising data raised in human RA implied macrophage mediators in disease progression [1,2]. Macrophages are the major source of inflammatory mediators during immune response once activated by auto-antibodies or by antigen-specific Th1 cell-derived lymphokines [2,3]. Though essential for the elimination of invasive antigens, chronic expression of the above mediators can induce a variety of inflammatory disorders, including RA and many other autoimmune diseases [2]. During RA, patients have an increased number of monocytes, particularly inflammatory monocytes, circulating in peripheral blood [4-6] and have an elevated number of macrophages in the joints [5]. These cells are highly activated and are one of the main producers of interleukin (IL)-1β and tumor necrosis factor-alpha (TNF-α), two essential proinflammatory cytokines required for the progression of RA because they are capable of inducing other proinflammatory cytokines and activating matrix metalloproteinases in autocrine and paracrine fashions [2]. Inhibitors of IL-1 and TNF-α cause a reduction in synovial inflammation, bone destruction, and macrophage infiltration in patients with RA [7,8]. A critical role of TNF-α and IL-1 during RA pathogenesis was confirmed by the recent development of appropriate therapeutic counterstructures [9].

In patients with autoimmune diseases, the use of dietary supplements is on the rise, mainly because they are effective, inexpensive, and relatively safe [10]. Recent studies indicate that two main flavonols, quercetin and its glycosylated form, rutin (or rutoside, RU), attenuate various inflammatory functions of macrophages in human or animal models [11-15]. Flavonols are compounds isolated from various plants that traditionally have been used for pain and vascular protection [11]. Quercetin inhibits inflammatory reactions by regulating the generation of inflammatory cytokines such as IL-6, TNF-α, and interferon-gamma and associated activation protein-1 (AP-1) and nuclear factor-kappa-B (NF-κB) signaling pathways in immune cells *in vitro *and *in vivo *[10,15]. RU has similar *in vitro *effects on immune cells but differs from quercetin by its higher therapeutic index and the absence of a modulatory effect on the cell cycle and apoptosis [16,17].

Various RU formulations for systemic use have been commercially available for more than 40 years and are used primarily as treatment for edema related to venous insufficiency [11]. Oral administration of RU attenuated bowel inflammatory syndrome [18] and a variety of other acute and chronic inflammations in murine models [19,20]. The scavenging property of rutin led to a decrease of oxygen radical overproduction of leukocytes of patients with RA *in vitro *[21]. Meanwhile, the exact anti-inflammatory mechanism(s) of RU and its cellular target(s) were not elucidated even though a decrease of nitric oxide (NO) and IL-1β production has recently been suggested in mice [19].

This led us to investigate the anti-inflammatory potential of RU on purified human activated macrophages, key effector cells in inflammatory diseases. Macrophage-related inflammatory responses were then analyzed at transcriptomic and proteic levels *in vitro *in order to clarify the anti-inflammatory effect of RU in human cells. RU was subsequently tested *in vivo *at preventive or postarthritic levels in a rat model of chronic arthritis. Data point out the inhibitory effect of RU on inflammatory cytokines, corroborating its ability to significantly reduce clinical signs in arthritic rats.

## Materials and methods

### Reagents

For *in vitro *experiments, 3,3',4',5,7-pentahydroxyflavone-3-rutinoside trihydrate (RU) (>97% purity powder; Acros Organics, Noisy-le-grand, France) was used after suspension in distilled water. For *in vivo *subcutaneous (s.c.) injections in rats, RU was suspended in saline.

### Human cells

Peripheral blood samples were obtained from healthy volunteers with their informed consent after the approval of this study by the institutional ethics committee. These samples were pretested for the absence of HIV or hepatitis virus infections. Peripheral blood-derived mononuclear leukocytes (PBLs) were obtained by Ficoll gradient separation, and monocytes were subsequently separated from other leukocytes by adherence to CD14 beads (Miltenyi Biotec, Paris, France). CD14^- ^PBLs were used for the hematotoxicity test of various RU preparations. Briefly, cells were incubated in medium alone or with 10^-6 ^M phytohemagglutinin-P (PHA) (5 μg/mL; Murex Biotech Ltd, Dartford, UK) in order to induce lymphocyte proliferation. RU was added at different concentrations, cells were harvested 4 days later, and apoptotic/necrotic versus total cells were counted as indicated below. CD14^+ ^cells were then suspended in RPMI 1640 medium supplemented with 100 U/mL penicillin, 100 μg/mL streptomycin, 2 mM glutamine, and 10% fetal calf serum (FCS) (all from Gibco-Europe, Paisley, Scotland). The above culture medium, chemicals, and FCS were endotoxin-free and tested for the absence of direct activation effects on human monocytes (CD23 expression and TNF-α production as activation markers). After these procedures, more than 95% of cells expressed CD14 antigen and displayed cytochemical characteristics of monocytes/macrophages [22].

### Experimental arthritis and rats

Female Lewis rats (Janvier, Le Genest St Isle, France) were housed under standard laboratory conditions with free access to food and water. The temperature was kept at 22°C ± 2°C, and a 12-hour light/dark schedule was maintained. The Animal Research Committee of the Agriculture Ministry approved this investigation. All animal procedures were performed in strict accordance with the guidelines issued by the European Economic Community (directive 86/609). Adjuvant-induced arthritis (AIA) was obtained in 6-week-old animals by s.c. injection at the base of the tail of 300 μL (1.8 mg) of inactivated *Mycobacterium butyricum *(Difco Laboratories Inc., now part of Becton Dickinson and Company, Franklin Lakes, NJ, USA) diluted in an emulsion of 8 mL of Vaseline oil, 1 mL of polysorbate 80, and 1 mL of phosphate-buffered saline (PBS) (Laboratoires Eurobio, Courtaboeuf, France). Rats were boosted 1 week later with the same dose of antigen and observed for up to 50 days after immunization for clinical signs of chronic arthritis. Evaluation of AIA severity was performed by two independent observers with no knowledge of the treatment protocol. The severity of AIA in each paw was quantified daily by an arbitrary clinical score measurement from 0 to 2 as follows: no signs of inflammation (0), swelling alone (0.5) for each paw, immobility (0.5) for each paw, 2 being the highest score with both paws swelling and immobile. Weight evolution of the animals was measured daily. Rats were treated with five injections (every 2 days) of RU, saline, or hydrocortisone (HC) (Sigma-Aldrich, Saint Quentin Fallavier, France) as indicated in the Results section. Injections were initiated 1 day after the appearance of the first arthritic symptoms (therapeutic) or on the first day of immunization (preventive). For macrophage collection, animals were anaesthetized with ether and the peritoneal cavity was washed with 10 mL of cold PBS (pH 7.4). After centrifugation at 300 *g *for 10 minutes at 4°C, cells were collected, counted, and adjusted to 10^6 ^cells per milliliter with culture medium. After 96-hour incubation in medium alone, cell supernatants from disease-free and AIA rats were collected for mediator quantification. To activate the production of various inflammatory mediators from rat peritoneal macrophages, cells were incubated with lipopolysaccharide (10 μg/mL; Sigma-Aldrich). RU was added simultaneously to cell activation. Cells or their supernatants were then tested for the presence of various inflammatory mediators.

### Human cell activation

Monocytes/macrophages were activated through the physiological CD23 pathway [22]. PBL-derived adherent cells must be preactivated to express surface CD23 as described [22] following their incubation with various cytokines, including recombinant human IL-4 (50 ng/mL), during 24 hours at 37°C. After washing, cells were tested for their surface CD23 expression (>80% CD23^+^) and then incubated in the presence of crosslinking CD23-MAb (clones 135, IgG1k, 20 μg/mL) during 48 to 96 hours or of IgE and anti-IgE as described [22,23]. Cells may then be analyzed for their NOx (nitrite/nitrate) content (see below) and cell supernatants for the presence of various inflammatory mediators. To detect cell apoptosis, externalization of inner membrane phosphatidylserine and DNA content was investigated by flow cytometry using a fluorescein-conjugated annexin V and propidium iodide kit (Immunotech, Marseille, France).

### RNA preparations and transcriptomic arrays

After cultures, total RNA was extracted using Trizol (Invitrogen, Cergy Pontoise, France) and was subsequently purified on RNeasy columns (Qiagen, Hilden, Germany). Synthesis of biotin-labelled cRNA, purification, and hybridization (6 μg) to custom array membranes were performed according to the manufacturer's recommendations (OHS-011; SuperArray Bioscience Corporation, Frederick, MD, USA). For detailed gene content and housekeeping controls, see reference [24]. After local background subtraction, average signal intensity from duplicate spots was compared with values obtained for housekeeping genes using Alpha Imager HP automatic image capture software (Alpha-Innotec, San Leandro, CA, USA). For each gene, modulation was defined as the relative expression value for stimulated versus control sample.

### Quantification of inflammatory mediators

For intracellular NO measurements, cells (>10^5^) were incubated with 10 μM DAF-FM-DA (4-amino-5-methylamino-2',7'-difluoro-fluorescein diacetate; Molecular Probes, now part of Invitrogen) for 1 hour at 37°C in 1 mL of culture medium. Cells were then washed in PBS and incubated in 0.5 mL of PBS for 30 minutes at 37°C. Intracellular NO content was then investigated by flow cytometry. Cell supernatants (48 to 72 hours) were assayed for the stable end product of NO, NO_2_^- ^using the Griess reaction modified as detailed elsewhere [25]. The inhibitory analog of L-arginine, N(6)-(1-iminoethyl)-L-lysine/2HCl (L-NIL) (Coger SA, Paris, France) [26], was used to inhibit inducible nitric oxide synthase (iNOS)-mediated NO generation at a concentration of 1 mM. To detect human cytokine levels, we have used a human multi-assay Th1/Th2 II plex kit (Bender MedSystems, Vienna, Austria) and flow cytometry. Inflammatory mediators in rat sera or cell supernatants were quantified using appropriate enzyme-linked immunosorbent assay kits in accordance with the manufacturer's recommendations for prostaglandin E_2 _(PGE_2_) (R&D Systems Europe, Lille, France), monocyte chemoattractant protein-1 (MCP-1) (Tebu, Le Perray-en Yveline, France), TNF-α, and IL-1β (Biosource, Montrouge, France).

### Statistical analysis

Comparisons were assessed using the Fisher exact test for proportions and the Mann-Whitney *U *test for quantitative values. A *p *value of less than 0.05 was considered significant. For some rat *in vitro *experiments, results were analyzed and compared using the Student *t *test for paired data.

## Results

### Inhibition of gene transcription in human macrophage by rutoside

**Figure 1:**
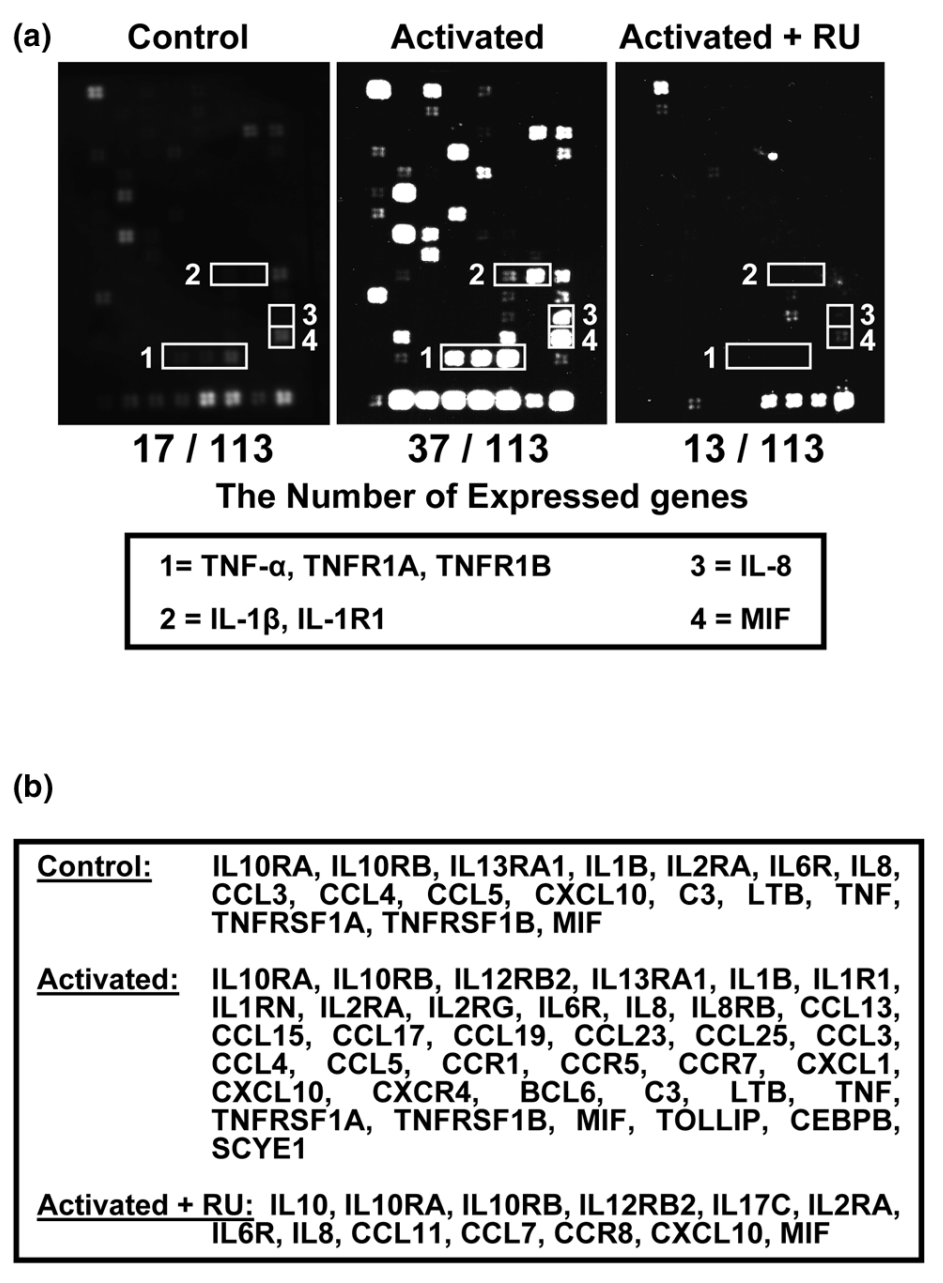
Rutoside (RU) mediates the inhibition of inflammatory gene transcription in activated human macrophages. Human monocyte-derived macrophages were activated (10 μg/mL CD23-McAb) alone or in the presence of 100 μM RU. After 24 hours of incubation, cellular mRNAs were extracted and analyzed by inflammation-specific macroarray. **(a) **RU inhibits gene expression by activated human macrophages, with the exception of interleukin (IL)-10. A representative array from two distinct experiments is shown. **(b) **Inflammatory genes detected on each membrane, in addition to controls. MIF, macrophage migration inhibitory factor; TNF, tumor necrosis factor.

*In vitro*, RU has generally been shown to display its activities at concentrations of 30 to 200 μM [19]. Prior to RU use, we first tested its effect on human normal or PHA-mediated cycling PBLs *in vitro*. We had no significant increase of the number of apoptotic (annexin^+^) or necrotic (propidium iodide^+^) cells after 96 hours of cell incubation in the presence of 1 to 200 μM RU (range from -5% to +7% of cells). RU was then used at concentrations of less than or equal to 100 μM in the following experiments. After their separation, human monocyte-derived macrophages were activated in the presence of 100 μM RU. The transcription of inflammatory genes was then analyzed using a macrophage-specific macroarray. Compared with resting cells, activated macrophages acquire a significant expression of 20 new mRNAs encoding various inflammatory mediators, chemotactic factors, and their receptors (Figure 1). In addition, we observed a significant increase in mRNA expression for genes encoding IL-1β, IL-8, TNF-α, TNF-R1, and macrophage migration inhibitory factor (MIF) (>110%; *p *< 0.05), known for their critical role during inflammatory response [2,27]. Treatment of macrophages with RU inhibited the expression of most of the above genes (19/20 were totally suppressed), including IL-1β, TNF-α, TNF-R1, and many chemoattracting factors, or decreased the expression of genes such as MIF and IL-8 (*p *< 0.05) (Figure 1). Surprisingly, the transcription of the gene encoding IL-10, known as Th2 type cytokine, was significantly increased (>230%; *p *< 0.05). These findings indicate that RU is a potent inhibitor of the transcription of proinflammatory genes in human macrophages with the exception of that encoding IL-10.

### Rutoside modulates the generation of inflammatory mediators from activated macrophages

**Figure 2:**
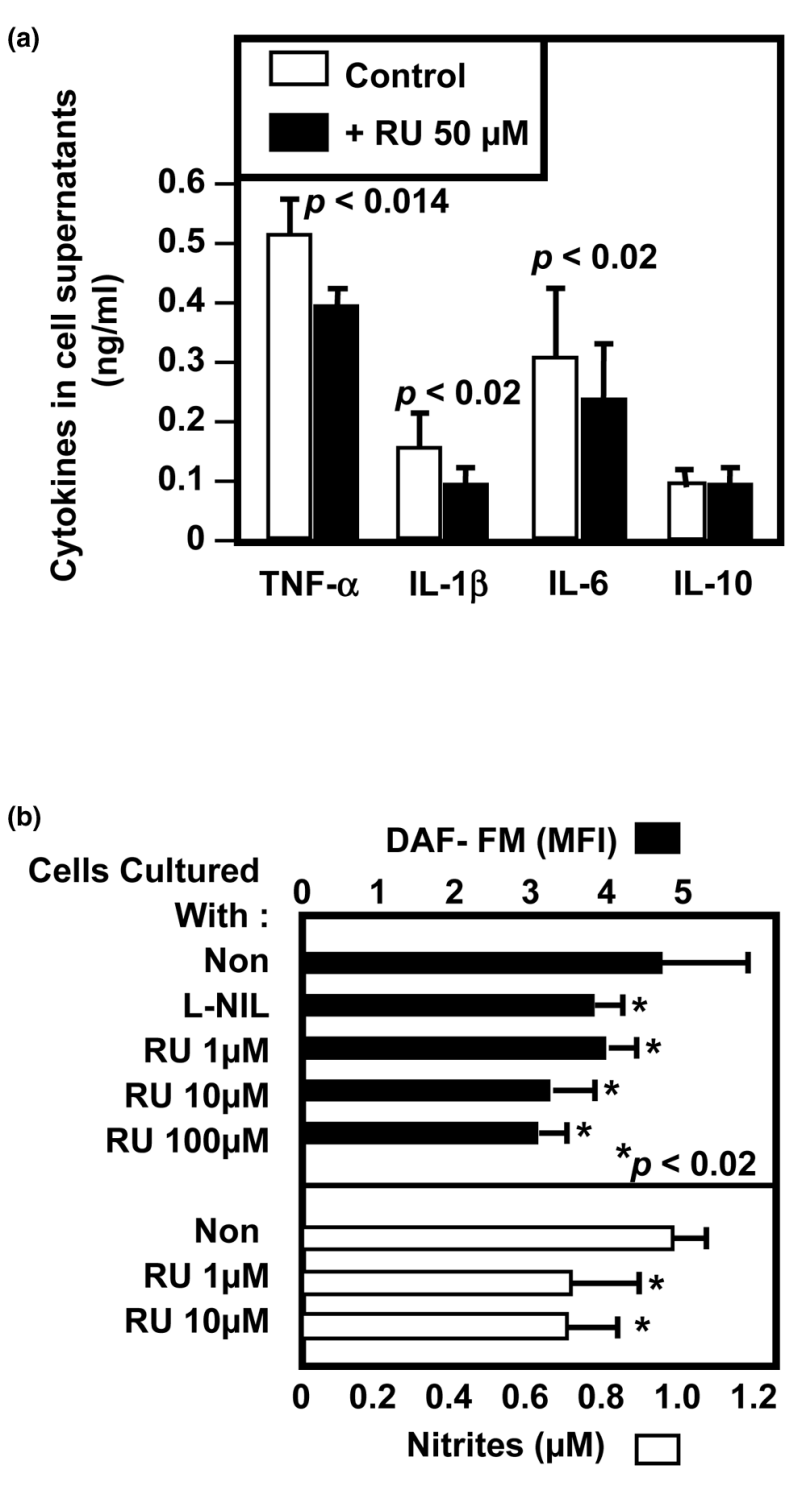
Inhibition of inflammatory mediator generation from activated human macrophages. **(a) **Rutoside (RU) decreased the generation of tumor necrosis factor-alpha (TNF-α), interleukin (IL)-1β, and IL-6 but not IL-10 from activated human macrophages. **(b) **Inhibition of nitric oxide (NO) generation from human activated macrophages after their incubation with various RU concentrations. RU decreases both intracellular NO (upper panel) and extracellular nitrites (lower panel). Specific inducible nitric oxide synthase inhibitor (L-NIL, 1 mM) was used as control. Results from three different cell preparations ± standard deviation are shown. *P value obtained as compared to activated cells. L-NIL, N(6)-(1-iminoethyl)-L-lysine/2HCl.

Transcriptomic data led us to investigate the effect of RU on cytokine production at the protein level. Quantification of various cytokines in macrophage cell supernatants indicates that the addition of RU to human activated macrophages significantly (*p *< 0.02) decreased the concentrations of TNF-α, IL-1β, and IL-6 (Figure 2a), three critical proinflammatory cytokines. We failed to detect significant modulation of IL-10 levels in contrast to mRNA data (Figures 1b and 2a).

In addition to the above cytokines, inflammatory macrophages produce various non-proteic mediators, including iNOS-dependent NO [22]. Upon their activation, human macrophages produce NO, detected at the intracellular level by specific probe (DF-FM) and at the extracellular level through the quantification of nitrites, the final metabolites of NO in cell supernatants. Data in Figure 2b show that RU partly inhibited NO generation in a dose-dependent manner at both the intracellular and extracellular levels. Generation of NO from human macrophages was also reversed by L-NIL (Figure 2b), a specific inhibitor of iNOS [26], suggesting the ability of RU to reverse iNOS-mediated NO production.

### Rutoside decreases and prevents arthritic signs in rats

**Figure 3:**
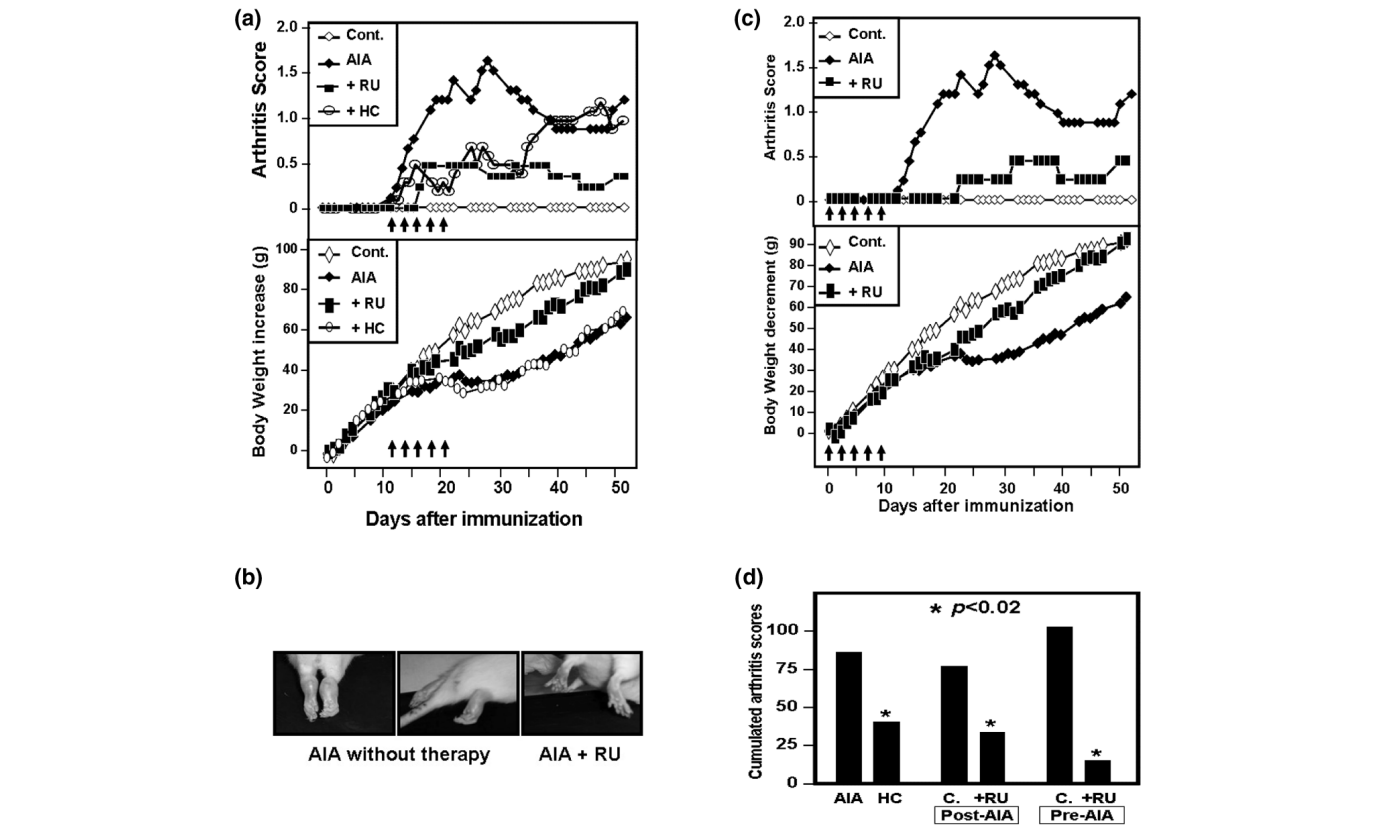
The evolution of arthritis severity scores of adjuvant-induced arthritis (AIA) in rats following their treatment with rutoside (RU). **(a) **Arthritis clinical scores (upper panel) and body weight (lower panel) of young AIA rats following their treatment with 133 mg/kg subcutaneous doses of RU suspension, every 2 days × 5, as indicated by arrows starting after the appearance of clinical symptoms. As positive control, AIA rats received five injections of 30 mg/kg hydrocortisone (HC). Results are means from five rats from each group (standard deviation [SD] less than 25% for all groups). **(b) **External aspects of rat paws showing swelling (left), swelling + immobility (central), and healed paws following RU treatment (right). **(c) **Amelioration of arthritis severity scores (upper panel) and body weight (lower panel) of AIA rats following preventive treatment with 5 × 133 mg/kg doses of RU suspension, starting the first day of immunization, before AIA development. Results are means from five rats (SD less than 25% for all groups). **(d) **Cumulative AIA clinical scores from untreated rats, hydrocortisone (HC)-treated rats, or those treated by RU prior to AIA development (Pre-AIA) or following arthritis development (Post-AIA). Bars represents cumulative AIA severity scores over the course of 40 days of observation (days 0 to 40) of five rats from each group. *P value compared to untreated AIA rats.

*In vitro *observations in human cells led us to investigate the therapeutic anti-inflammatory effect of RU *in vivo *in rat AIA, an experimental model with many clinical and histopathological features of chronic human RA [28]. RU formulations and doses used in this work were based upon various *in vivo *studies [29] and our preliminary analysis showing the absence of an apparent toxic effect of up to 3,000 mg/kg total doses (data not shown). AIA Lewis rats thus were treated with RU suspension at 133 mg/kg s.c. doses (one dose every 2 days × 5). As therapeutic positive control, rats were treated with HC at 150 mg/kg total dose [30]. We found that administering five doses of RU, beginning the day after the appearance of the first arthritic symptoms, significantly improved the clinical course, including arthritic scores and weight progression of AIA rats as compared with the control groups (Figure 3a; *p *< 0.0001). The effect of RU was superior to that of HC in inhibiting arthritic scores (*p *< 0.001) and remained stable after treatment (Figure 3a). Figure 3b shows the external aspect of inflamed pads in RU-treated rats compared with untreated rats. No obvious toxicity was observed resulting from the treatment in the rats (for example, the weight of the treated normal rats was close to untreated controls). Hence, these experiments indicate that the RU effectively ameliorates AIA.

In a second set of experiments, we tested the ability of RU to prevent AIA establishment in rats. We injected RU every 2 days for a total of five injections, beginning on the day of adjuvant injection to the rats and before the appearance of any arthritic signs. Notably, we found that pretreatment with RU could significantly reduce the severity of arthritis and growth delay observed in control groups (Figure 3c; *p *< 0.0002). Hence, as shown in Figure 3d, RU treatment was effective either at disease onset or prevention of severe disease establishment, whereas no such activity was obtained with controls.

### Rutoside and rat macrophage inflammatory mediators *ex vivo*

**Figure 4:**
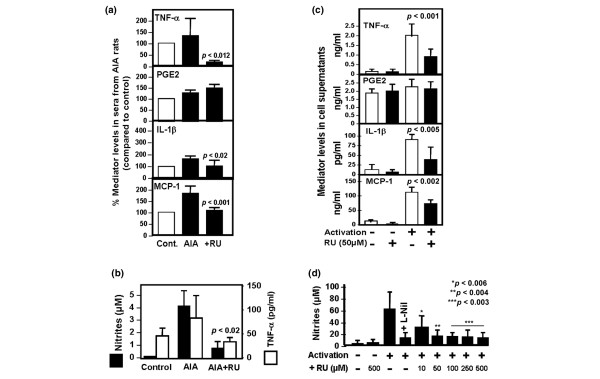
Effect of rutoside (RU) treatment on rat inflammatory mediators. **(a) **Sera were collected at day 50 after immunization and tested for their concentrations in tumor necrosis factor-alpha (TNF-α), prostaglandin E_2 _(PGE_2_), interleukin-1-beta (IL-1β), and monocyte chemoattractant protein-1 (MCP-1). Results are shown as mean percentage of modulation of cytokine levels compared to healthy rats. Mean percentage + standard deviation from three rats in each group is shown. *P *value compared to untreated adjuvant-induced arthritis (AIA) rats. **(b) **Freshly isolated peritoneal macrophages from untreated or RU-treated rats were collected on day 50 after immunization. They were incubated in medium alone during 48 hours, and their supernatants were collected and tested for their TNF-α or nitrite levels. *P *value compared to untreated AIA rat cells. **(c) **Macrophages from normal rats were incubated in medium alone or activated by lipopolysaccharide. RU was added (50 μM) simultaneously to cell activation. Cell supernatants were harvested 48 hours after incubation and tested for their concentrations of TNF-α, PGE_2_, IL-1β, and MCP-1. *P *value compared to activated cells. **(d) **RU inhibits the release of nitric oxide from activated rat macrophages in a dose-dependent manner. Bars show mean + standard deviation from three different rat cell preparations. *P *value compared to activated cells.

To confirm the anti-inflammatory effects of RU and the role of macrophages, we tested the levels of selected mediators in the sera from AIA animals following their treatment with or without RU. Data (Figure 4a) show that RU significantly reduced the levels of TNF-α, IL-1β, and MCP-1 in animal sera (*p *< 0.02), whereas the level of PGE_2_, an indirect marker of COX-2 (cyclooxygenase-2) activity, was not affected by this treatment. These data reinforced human findings as they revealed that RU decreased the level of inflammatory cytokines, critical for RA pathogenesis *in vivo*. Although known as a major source of the above mediators [2], the role of macrophages during RU treatment required more direct *ex vivo *analysis. Peritoneal macrophages were then isolated from RU-treated or untreated arthritic rats and promptly incubated in medium alone. After 48 hours of incubation, cell supernatants were analyzed for the levels of TNF-α and nitrites as inflammatory markers. Our results (Figure 4b) clearly show that increased inflammatory macrophage-derived TNF-α and NO in AIA rats were attenuated after treatment with RU.

Finally, we tested the ability of RU to reduce rat macrophage inflammatory responses *in vitro*. Peritoneal macrophages were isolated from healthy rats and activated without various doses of RU. Our data show (Figure 4d) that the levels of NO, as indicated by the concentration of nitrites in cell supernatants, decreased after the addition of RU in a dose-dependent manner (*p *< 0.006). As in human cells, L-NIL had a similar inhibitory effect compared with RU, suggesting its ability to downregulate iNOS-mediated NO generation in macrophages. Inhibition reached a plateau at 50 μM RU, a concentration that was subsequently used to investigate the RU effect on other mediators. As in *in vivo *findings, RU decreased the level of inflammatory cytokines (TNF-α, IL-1β, and MCP-1) generated from activated rat macrophages (Figure 4c). As in *ex vivo *data, the production of PGE_2 _was not modified after the addition of similar RU dilution.

## Discussion

Macrophages arise as an interesting target for modulation of inflammatory disease. Inflammatory mediators derived from these cells have a critical role during synovial inflammation and bone destruction in some patients with RA [7,8]. Obtained using various approaches, our results clearly indicate that RU, a molecule already used in vascular diseases, inhibited the activation of human macrophages and the subsequent secretion of proinflammatory mediators from these cells. RU was shown to inhibit the transcription of more than 20 genes encoding critical proinflammatory factors, including TNF-α, IL-1, IL-8, TNF-α, MIF, and chemoattracting factors. This effect was confirmed by decreased concentrations of IL-1β, TNF-α, and IL-6 observed in cell supernatants.

NO has been identified as another proinflammatory mediator in human arthritis and experimental animal studies [3,31]. Increased concentrations of nitrites, stable metabolites of NO, have been observed in the serum and the synovial fluid of patients with RA and osteoarthritis [3,32]. Increased iNOS activity and NO production have also been detected in the blood mononuclear cells of patients with RA and correlated with the tender and swollen joint counts [33]. Here, we show that RU decreased the production of iNOS-mediated NO by human macrophages in a dose-dependent manner. Of particular interest, and by contrast to the above mediators, RU induced a slight but significant increase of IL-10 mRNA. However, we failed to detect an increase of IL-10 protein level. This may be due to the absorption of IL-10 by macrophages, as yet observed in CD23-activated human epithelial cells [34]. This Th2 type cytokine is well known as a downregulator of the above-mentioned Th1 mediators in human macrophages [35]. These preliminary data suggest that RU preferentially lowers Th1-like cytokine generation from human macrophages.

To support the therapeutic interest of RU, we investigated its effects in a rat model of adjuvant-induced chronic arthritis, well known to mimic Th1 type pathogenic signs of RA [28]. RU significantly reversed growth delay and severe AIA development in rats with persistent partial or complete long-term recovery, not observed in rats treated with HC. These data confirm early observations in experimental acute inflammation models [18-20,36] and further revealed the preventive property of RU in arthritic rats. *Ex vivo *analysis of rat sera and macrophages confirmed RU-mediated inhibition of critical proarthritic factors such as TNF-α, IL-1, MCP-1, and NO *in vivo*. This property correlated with RU-mediated inhibition of murine macrophage activation and inflammatory mediator release *in vitro *(Figures 3 and 4). In contrast to quercetin [37], RU did not mediate the inhibition of the PGE_2 _pathway.

Together, the above data provide new insight into the possible mechanism of anti-inflammatory effects of RU in macrophages. Human and murine analyses of cytokine expression clearly show that RU suppresses the major inflammatory and proarthritic mediators of macrophages. The ability of RU to decrease MCP-1 levels *in vivo *and *in vitro *may add to its beneficial effects because this cytokine is a potent stimulator of monocyte recruitment into the site of inflammation [38]. We have previously shown that the nonglycosylated derivative of RU, quercetin, inhibits the production of TNF-α and NO from activated human macrophages [39]. These flavonols inhibit the phosphorylation and activation of Jun N-terminal kinase/stress-activated protein kinase, leading to the suppression of AP-1 activation. They also decrease the activation of NF-κB in both human and experimental models [12,40]. These observations may explain the anti-inflammatory property of RU because both nuclear factors are necessary for the generation of most inflammatory mediators analyzed in the present study [41-43].

However, the exact mechanism underlying the improvement in the arthritis model requires more experimental clarifications. Despite the direct relationship between arthritis signs and macrophage inflammatory markers in the AIA rat model, we must not exclude the simultaneous effect of RU on other inflammatory partners (such as lymphocytes) that may directly or indirectly reduce arthritis manifestations. In addition, AIA is a good model for Th1-mediated and macrophage inflammatory response but is a poor model for Th2-mediated immune reactions. Further analysis of RU activities in the presence of various human lymphocyte populations is required to clarify these points.

Finally, *in vivo *use of quercetin as medicine suffers from the lack of approved formulation despite a first preliminary assay as adjunct treatment of prostatitis/chronic pelvic pain in humans [44]. In contrast, formulations containing either RU or its derivatives are currently used in the treatment and prevention of venous circulation disorders [11,45].

## Conclusion

RU appears as an interesting cost-effective therapeutic tool in inflammatory diseases and represents an alternative to immunosuppressor agents well known for their multiple side effects.

## Abbreviations

AIA = adjuvant-induced arthritis; AP-1 = activation protein-1; FCS = fetal calf serum; HC = hydrocortisone; IL = interleukin; iNOS = inducible nitric oxide synthase; L-NIL = N(6)-(1-iminoethyl)-L-lysine/2HCl; MCP-1 = monocyte chemoattractant protein-1; MIF = macrophage migration inhibitory factor; NF-κB = nuclear factor-kappa-B; NO = nitric oxide; PBL = peripheral blood-derived mononuclear leukocyte; PBS = phosphate-buffered saline; PGE_2 _= prostaglandin E_2_; PHA = phytohemagglutinin-P; RA = rheumatoid arthritis; RU = rutoside; s.c. = subcutaneous; TNF-α = tumor necrosis factor-alpha.

## Competing interests

The authors declare that they have no competing interests.

## Authors' contributions

TK contributed to the acquisition of data in human and murine cells and *in vivo*. DM contributed to the conception and design of the study and to manuscript drafting. JR and AA-K contributed to the acquisition of data in human cells. SB and DT contributed to the acquisition of data *in vivo*. RE contributed to the analysis and interpretation of data. FF contributed to toxicological analysis and interpretation of data. MDM contributed to the conception and design of the study, analysis and interpretation of data, and manuscript drafting. All authors read and approved the final manuscript.
